# Procedural confidence in hospital based practitioners: implications for the training and practice of doctors at all grades

**DOI:** 10.1186/1472-6920-9-2

**Published:** 2009-01-12

**Authors:** Rona M Connick, Peter Connick, Angelos E Klotsas, Petroula A Tsagkaraki, Effrossyni Gkrania-Klotsas

**Affiliations:** 1 Department of Acute Medicine, Hinchingbrooke Hospital, Hinchingbrooke Health Care NHS Trust, Hinchingbrooke, UK; 2 Department of Clinical Neurosciences, University of Cambridge, Cambridge, UK; 3 Department of Medicine, Ipswich Hospital, The Ipswich Hospital NHS Trust, Ipswich, UK; 4 Department of Infectious Diseases, Addenbrooke's Hospital, Cambridge University Hospitals NHS Foundation Trust, Cambridge, UK

## Abstract

**Background:**

Medical doctors routinely undertake a number of practical procedures and these should be performed competently. The UK Postgraduate Medical Education and Training Board (PMETB) curriculum lists the procedures trainees should be competent in. We aimed to describe medical practitioner's confidence in their procedural skills, and to define which practical procedures are important in current medical practice.

**Methods:**

A cross sectional observational study was performed measuring procedural confidence in 181 hospital practitioners at all grades from 2 centres in East Anglia, England.

**Results:**

Both trainees and consultants provide significant service provision. SpR level doctors perform the widest range and the highest median number of procedures per year. Most consultants perform few if any procedures, however some perform a narrow range at high volume. Cumulative confidence for the procedures tested peaks in the SpR grade. Five key procedures (central line insertion, lumbar puncture, pleural aspiration, ascitic aspiration, and intercostal drain insertion) are the most commonly performed, are seen as important generic skills, and correspond to the total number of procedures for which confidence can be maintained. Key determinants of confidence are gender, number of procedures performed in the previous year and total number of procedures performed.

**Conclusion:**

The highest volume of service requirement is for six procedures. The procedural confidence is dependent upon gender, number of procedures performed in the previous year and total number of procedures performed. This has implications for those designing the training curriculum and with regards the move to shorten the duration of training.

## Background

Medical doctors routinely undertake a number of practical procedures and these should be performed competently [1,2]. Knowing which procedures are required in daily practice is important for those involved in planning training programmes and for accreditation bodies. However the challenge to achieve and maintain procedural competence for all clinicians is significant, with high volume alone no longer accepted as an adequate guarantee of competency. In addition there has been a recent decline in the total number of procedures performed by hospital general medicine practitioners [3]. and a drive to shorten the average duration of medical training despite continued widespread perception that procedural competency is important[4].

The new UK Postgraduate Medical Education and Training Board (PMETB) curriculum introduced in October 2007 expanded the previous list of specific procedures that the trainee is expected to be competent in by the end of core medical training. The new requirements are:

• Venepuncture

• Cannula insertion including large bore

• Arterial blood gas sampling

• Lumbar puncture*

• Pleural tap and aspiration*

• Intercostal drain insertion (Seldinger technique)*

• Ascitic tap*

• Abdominal paracentesis*

• Central venous cannulation*

• Initial airway protection: chin lift, guedel airway, nasal airway, laryngeal mask

• Basic and subsequently advanced cardio respiratory resuscitation.

• DC cardioversion*

• Urethral catheterisation

• Nasogastric tube placement

• Electrocardiogram

• Knee aspiration*

• Temporary cardiac pacing by internal wire or external pacemaker*

• Skin biopsy (this is not mandated for all trainees but opportunities to become competent in this technique should be available especially for trainees who subsequently wish to undertake specialist dermatology training)

The procedures common between the previous and new requirements are noted with asterisks. The previous version also included the use of continuous positive airway pressure (CPAP) and bilevel positive airway pressure (BiPAP) support, as well as caring for an existing tracheostomy. These are omitted from the new training curriculum.

Achieving initial procedural competence is the subject of numerous training initiatives including a change from the traditional bedside "see-one, do-one" approach to that involving initial skill-lab and subsequent decreasing levels of supervised support at the bedside. However, maintaining competence may also require significant effort, particularly in an environment where the total number of procedures performed is decreasing. There is a complex relationship between procedural competence and confidence. Confidence can be used as a marker of competence but the correlation is poor [5-7]. However, procedural confidence is of intrinsic importance through influence on the practitioner's willingness to undertake procedures, accurate self-assessment of their skills, and willingness to ask for support [8]. Procedural confidence also independently affects performance and is *per se *an important target for maintaining competency [9].

Furthermore, practitioners should not be required in their routine practice to perform procedures for which they do not feel confident of their own competence, as this would breach of the principles of Good Medical Practice.

The level of confidence amongst hospital practitioners to perform these "key" procedures is unknown. Clearly, it is unrealistic to expect all doctors to perform all procedures with competence and confidence at all levels of training, however guidance on what would represent an acceptable standard is limited. No specific standards are available for consultants although it is expected that those taking part in the medical rota should be able to assist a trainee should an emergency arise, themselves supported by relevant specialists (anaesthetists, cardiologists, interventional radiologists etc.). The UK Government Department of Health has identified continuing professional development and training as crucial to achieving high quality patient care [10]. However current NHS consultant appraisal documentation makes no special provision to demonstrate continuing competence in performing medical procedures [11]. The current and previous systems of UK medical training are shown in supplementary figure 1 . Specialist registrars ([SpR] equivalent to ST3 and above) are required be competent in performing these procedures as well as instruction, appraisal and assessment of junior doctors' performance. Senior house officers ([SHO] equivalent to ST1 and ST2) are required to gain experience of these procedures in order to attain independent competence. Foundation Year 1 (F1) doctors are required to be competent and confident to perform, and competent to teach undergraduates on; venepuncture and cannulation, arterial puncture, blood culture from central or peripheral sites, subcutanous, intradermal, intramuscular and intravenous injections, IV medication preparation and administration, performing ECG, spirometry and peak flow, urethral catheterisation, airway care (including simple adjuncts), and nasogastric tube insertion. Foundation year 2 (FY2) doctors are expected to maintain and improve their skills in the above procedures and expend the range of procedures they do such as aspiration of pleural fluid or air, skin suturing, lumbar puncture, insertion of a central venous line and aspiration of joint effusion [12].

We primarily set out to describe existing levels of procedural confidence. As secondary aims we set out to describe the factors affecting procedural confidence, and to assess the wider views on whether these procedures should be core competencies for all doctors. A cross-sectional questionnaire survey of hospital practitioners in East Anglia, UK was undertaken.

## Methods

A cross sectional study was performed of hospital doctors working in East Anglia, England. Two centres were involved; Addenbrooke's Hospital (Cambridge) – a 1,100 bed teaching hospital providing acute and specialist services for the local (≈0.5 M) and regional (≈2.5 M) population, and Ipswich Hospital (Ipswich) – a 317 bed teaching hospital providing acute services for the local population (≈0.2 M).

Anonymised questionnaires were distributed to all doctors holding a permanent position at both centres. Members of staff on temporary contracts were directly approached *ad hoc *either on the wards or in doctor's mess areas. No ethical approval was deemed necessary for the above study. The audit departments of both hospitals approved the audit activity.

Responders were asked a total of sixty-six questions across eleven domains. The first domain captured responder characteristics of gender, specialty (medical/surgical/other), training grade (FY1, FY2, SHO, SpR/ST, Consultant, Staff Grade/Clinical Fellow), year of graduation from medical school, number of years in current grade, number of years in medical practice since graduation, and total number of years in medical practice including medical research since graduation. The remaining ten domains addressed procedure-related competency for lumbar puncture, intercostal drain insertion and management, central venous line insertion, temporary cardiac pacing, elective DC cardioversion, pleural fluid aspiration, ascitic fluid aspiration, use of bilevel positive airways pressure (BiPAP) and continuous positive airways pressure (CPAP), and tracheostomy management respectively. The same 6 items were asked for each procedure. These were; total number of procedures performed throughout career, number of procedures performed in the past 12 months, and four forced choice questions. Responders were asked:

• How confident they currently felt to perform the procedure on a Likert rating scale with options of "very confident", "confident", "not so confident" or "not at all confident"

• How they were originally trained to perform the procedure with options of "see one do one (observing first hand)", "read about procedure", "internet based training", "clinical skills lab/simulation" or "other" with optional free text.

• If they thought that they needed further training to perform the procedure with options of "yes" or "no, I am fully competent to do it safely and accurately".

• If they thought that performing this procedure was important for all doctors to be able to perform competently with options of "yes" or "no, I think that only a few doctors need to be skilled in this procedure".

Statistical analysis was performed using Stata/SE 9.2 for Macintosh (Stata Corp. TX, USA).

## Results

Two hundred and eighty six forms were distributed. One hundred and eighty one (63%) were completed and returned, all are included in the subsequent analyses. Ascertainment was higher from the teaching hospital compared to the district general hospital (67% [133/200] *vs. *56% [48/86]). A response was given in ninety-two percent (3365/3662) of all questions asked. Form completion was higher from the teaching hospital group compared to the district general hospital (96% [2173/2261] *vs. *74% [606/816]). This difference is attributable to the completion of questions regarding responder characteristics (96% [891/931] *vs. *53% [178/336]). Completion of questions regarding procedural confidence scores is comparable between the two centres (96% [1282/1330] *vs. *97% [351/432]).

Forty one percent (75/181) of responders were female with no significant difference between centres (χ^2 ^test, p = 0.1229). Thirty two percent of responders specialised in internal medicine, twenty five percent in other clinical specialties, six percent in surgery and four percent in non-clinical specialties. The largest group of responders were specialist registrars or consultants within the first decade of appointment (table 1). The major service requirement over the previous year was for six procedures; central line insertion, lumbar puncture, pleural aspiration, ascitic aspiration, CPAP and intercostal drain insertion. Other procedures are performed less frequently. Most procedures are undertaken by senior house officers, specialist registrars and clinical fellows with members of each of these grades likely to have been called upon to perform central line insertion, lumbar puncture, pleural aspiration, ascitic aspiration and intercostal drain insertion at least once within the previous year (table 2). Despite the high service requirement for continuous positive airway (CPAP)/Bilevel positive airways pressure (BiPAP) support, this need is largely met by a few individuals. General clinicians do not provide a significant overall service input at any grade.

**Table 1:** Responder Characteristics

	**Addenbrooke's**	**Ipswich**	**Total**
	
**Gender (F:M)**	59:74	14:25	73:99
**Years of Clinical Practice**			
Median	9	6	8
Range	0–40	1–35	0–40
Lower Quartile	5	2	4
Upper Quartile	14	12	13
**Grade (percent)**			
FY1	4 (3)	6 (15)	10 (6)
FY2	7 (5)	4 (10)	11 (6)
SHO	11 (8)	9 (23)	20 (12)
SpR	43 (32)	12 (31)	55 (32)
Consultant	54 (41)	8 (21)	62 (36)
Clinical Fellow	14 (11)	0 (0)	14 (8)

Addenbrooke's = Addenbrooke's Hospital, Ipswich = Ipswich Hospital, FY1 = Foundation year 1, FY2 = Foundation year 2, SHO = Senior house officer, SpR = Specialist registrar

**Table 2:** Number of procedures performed in previous year

	**Central line insertion**	**Lumbar puncture**	**Pleural aspiration**	**Ascitic aspiration**	**CPAP**	**Intercostal drain insertion**	**Tracheostomy management**	**Knee joint aspiration**	**DC cardio-version**	**Temporary pacing**
	
**Cumulative number performed in previous year**	1266	561	758	677	648	633	366	271	218	71
**Grade**										
***FY1/FY2***										
**Median**	**0**	**1**	**1**	**0**	**0**	**0**	**0**	**0**	**0**	**0**
(95% CI)	(0 – 0)	(0 – 1.5)	(0 – 1.5)	(0 – 1)	(0 – 0)	(0 – 1)	(0 – 0)	(0 – 0)	(0 – 0)	(0 – 0)
Lower quartile	0	0	0	0	0	0	0	0	0	0
Upper quartile	0	2.5	2	1.5	0	1	0	0.5	0	0
***SHO***										
**Median**	**1**	**4.5**	**4.5**	**2**	**0**	**2**	**0**	**0**	**0**	**0**
(95% CI)	(0 – 4.9)	(2 – 10)	(1.1 – 9.8)	(0 – 5)	(0 – 1.9)	(0.1 – 4)	(0 – 1.3)	(0 – 0)	(0 – 2.8)	(0 – 0)
Lower quartile	0	2	1	0	0	0	0	0	0	0
Upper quartile	5	13.75	10	5	2.75	4	1	0.75	3	0
***SpR***										
**Median**	**6**	**4**	**4**	**1**	**0**	**3**	**0**	**0**	**0**	**0**
(95% CI)	(4 – 10)	(2 – 5.3)	(1.9 – 5)	(0 – 4.1)	(0 – 2.1)	(1 – 5)	(0 – 0)	(0 – 0)	(0 – 0)	(0 – 0)
Lower quartile	0	2	0	0	0	0	0	0	0	0
Upper quartile	15	10	10	5.5	8	10	1.75	0	1	1.25
***Consultant***										
**Median**	**0**	**0**	**0**	**0**	**0**	**0**	**0**	**0**	**0**	**0**
(95% CI)	(0 – 0)	(0 – 0)	(0 – 0)	(0 – 0)	0 – 0	(0 – 0)	(0 – 0)	(0 – 0)	(0 – 0)	(0 – 0)
Lower quartile	0	0	0	0	0	0	0	0	0	0
Upper quartile	2	1	1	0	0	1.75	1.25	0	0	0
***Clinical Fellow***										
**Median**	**3**	**3**	**5**	**4**	**1**	**1.5**	**0**	**0**	**0**	**0**
(95% CI)	(0 – 12)	(1.8 – 11.7)	(0 – 8.3)	(0 – 20)	(0 – 2.3)	(0 – 5.7)	(0 – 0.2)	(0 – 3.2)	(0 – 0)	(0 – 0)
Lower quartile	0	1.75	0	0	0	0	0	0	0	0
Upper quartile	12	12.5	8.5	20	2.5	6	0.25	3.25	0	0
***All***										
**Median**	**0**	**2**	**1**	**0**	**0**	**0**	**0**	**0**	**0**	**0**
(95% CI)	(0 – 2)	(1 – 2.8)	(0 – 2)	(0 – 0.1)	(0 – 0)	(0 – 1.3)	(0 – 0)	(0 – 0)	(0 – 0)	(0 – 0)
Lower quartile	0	0	0	0	0	0	0	0	0	0
Upper quartile	10	5	5	4	2	4	0.25	0	0	0

CPAP = continuous positive airway pressure support (responses also included bilevel positive pressure airway support), DC Cardioversion = elective direct current cardioversion, FY1 = Foundation year 1, FY2 = Foundation year 2, SHO = Senior house officer, SpR = Specialist registrar

**Figure 1:**
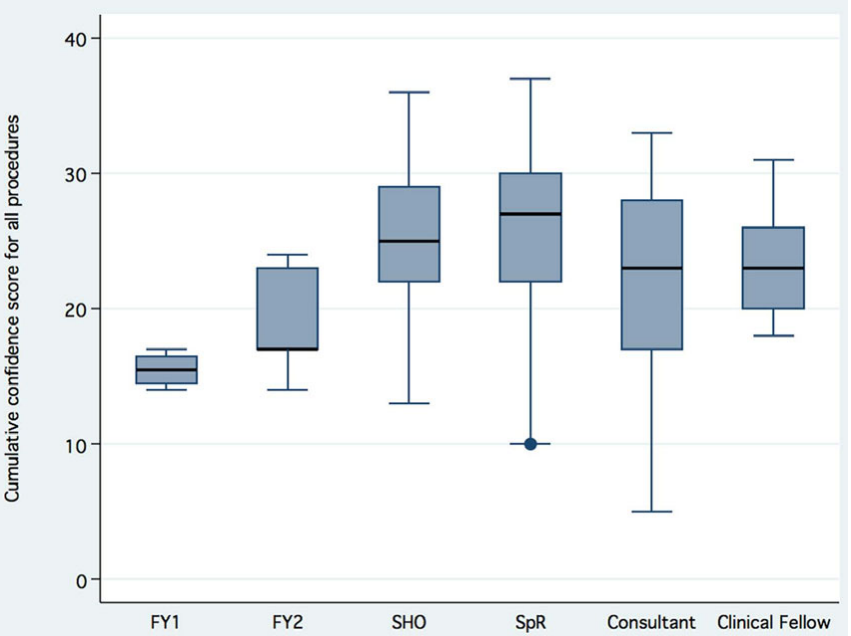
**Cumulative confidence score by grade**. FY1 = Foundation year 1, FY2 = Foundation year 2, SHO = Senior house officer, SpR = Specialist registrar.

**Figure 2:**
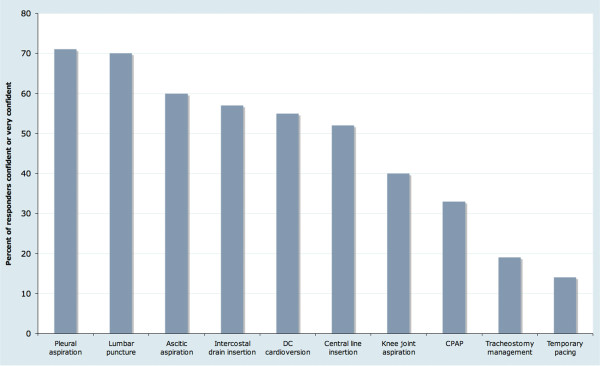
**Percent of responders "confident" or "very confident" by procedure**. CPAP = continuous positive airway pressure support (responses also included bilevel positive pressure airway support), DC Cardioversion = elective direct current cardioversion, FY1 = Foundation year 1, FY2 = Foundation year 2, SHO = Senior house officer, SpR = Specialist registrar.

Procedural confidence responses showed an "all or nothing" effect. Twenty seven percent (522/1762) of responses were "not at all confident", twenty seven percent (474/1762) of responses were "very confident", twenty one percent (376/1762) of responses were "confident" and nineteen percent (330/1762) of responses were "not so confident". This effect was seen for all procedures (supplementary figure 2 ). The individual cumulative confidence score ("not at all confident" = 1, "not so confident" = 2, "confident" = 3, "very confident" = 4; giving a cumulative confidence score range of 10 – 40) shows the trend that confidence for these ten procedures peaks in the specialist registrar grade, and declines in consultant practice (figure 1). Overall, clinicians at any grade are "confident" or "very confident" to perform 4 – 5 procedures, although this also exhibits variation by grade similar to that for cumulative confidence scores (table 3). The procedures which >50% responders felt "confident" or "very confident" to perform are lumbar puncture, pleural aspiration, ascitic aspiration, intercostal drain insertion, DC cardioversion and central line insertion. Few responders were confident in CPAP management or temporary cardiac pacing (figure 2).

**Table 3:** Number of procedures that responders are confident to perform by grade

	**Median (95% CI)**	**Lower quartile**	**Upper quartile**	**Range**
	
**FY1/FY2**	**1** (1 – 2)	1	3	0 – 5
**SHO**	**5** (4 – 6)	4	6	1 – 9
**SpR**	**7** (5.7 – 7)	4	7	1 – 10
**Consultant**	**4.5** (3.8 – 5)	2	7	0 – 10
**Clinical Fellow**	**4.5** (3.8 – 6)	3.75	6	2 – 7
**All**	**5** (4 – 5)	3	7	0 – 10

FY1 = Foundation year 1, FY2 = Foundation year 2, SHO = Senior house officer, SpR = Specialist registrar

Logistic regression analysis shows an independent effect on procedural confidence for gender (males having higher confidence), number of procedures performed in the previous year (each performed increasing confidence) and the total number of procedures performed – although the effect of procedures performed more than 1 year previously is small (table 4).

**Table 4:** Factors determining procedural confidence

	**Odds Ratio**	**OR 95% CI**	***p***
	
**Gender**	**1.39**	1.04 – 1.85	0.026
**Procedures performed in past year**	**1.29**	1.21 – 1.37	<0.001
**Procedures performed throughout career**	**1.01**	0.01 – 1.02	0.002
**Years of clinical practice**	**1.0**	0.98 – 1.01	0.674
**"Skills lab" based training**	**1.02**	0.52 – 2.0	0.964

The perceived need for further procedural training mirrors the changes in cumulative procedural confidence score by grade, with the perceived need lowest in the specialist registrar grade (the point of maximum cumulative procedural confidence scores) and increasing in consultant practice (supplementary figure 3 ). However, the perception that procedural competency is important for all practitioners declines throughout training, plateauing in the specialist registrar and consultant grades where only 45% of responses identified a procedure as a key generic skill (supplementary figure 4 ). Responses from specialist registrar or consultant grade identified five procedures which are viewed as important for all practitioners to be competent in; pleural aspiration (61%), lumbar puncture (59%), intercostal drain insertion (56%), ascitic aspiration (54%) and central line insertion (50%). The proportion of responses identifying the other five procedures as important generic skills is low; DC cardioversion (41%), knee joint aspiration (30%), CPAP support (20%), tracheostomy management (19%) and temporary pacing (14%).

## Discussion

This study was a cross sectional survey of confidence to perform practical procedures in hospital practitioners. We believe it to be the first description of procedural confidence levels in UK doctors at all grades. A doctor's confidence is important because it impacts on their willingness to undertake procedures, ask for support, and the self-assessment of their skills.^8 ^Procedural confidence also independently affects performance and is therefore an important target for maintaining competency [9]. Furthermore, doctors should not be required by their routine practice to perform procedures for which they do not feel confident of their own competence, as this would breach the principles of Good Medical Practice. The procedures chosen for this study reflected the PMETB medical curriculum requirements at the time of design. Ascertainment was comparable to similar studies using questionnaire based data capture. However, as with all self-reporting observational studies, systematic bias due to self-selection cannot be excluded. In addition, data on the number of procedures performed is subject to recall inaccuracy.

The management of hospital in-patients inevitably requires practical procedures to be performed. The service workload in this study sample was split equally between consultants (2729 procedures per year) and doctors in training (2576 procedures per year), although this is likely to underestimate the contribution of trainees as ascertainment from doctors below SpR/ST3 grade was low. Nevertheless, it is clear that both consultants and trainees make a significant contribution to the service requirement. However, there is a marked difference in the dispersion of procedural workload within these two groups. The consultant group's procedural workload was performed by few individuals (the majority of responders performing none of these procedures in the previous year), in high volume, and for only a very limited range of specific procedures per individual responder. In contrast, almost all trainees had performed procedures in the previous year, with the majority performing relatively low numbers (< 1 per month), and performing a wider range of specific procedures. It is not clear if this difference is due to trainees attempting to meet their PMETB requirement for competency in all 10 procedures, or expediency because trainee doctors are available when practical procedures are required. There is an apparent tension between the service requirement to perform these procedures, and the reality that skills acquired in doing so will be largely unnecessary at the consultant grade.

Confidence scores for all procedures were bimodal, with the majority of responders either "not at all confident" or "very confident". This is an unusual distribution of responses from a Likert scale and supports a "threshold effect" where doctors perceive themselves as not at all confident to perform a procedure until an internal threshold is reached triggering high levels of confidence [13]. This may reflect concerns about complications and the ethical principle of non-maleficence. As expected with the distribution of workload, cumulative confidence for all ten procedures peaks in the SpR grade then falls as a consultant. Nevertheless, only two responders (1%) were "confident" or "very confident" in all 10 procedures (one senior SpR and one newly appointed consultant). Clearly, almost all trainees fail to achieve the PMETB curriculum requirements. Six procedures emerged as the most commonly performed; central line insertion, lumbar puncture, pleural aspiration, ascitic aspiration, CPAP and intercostal drain insertion. Given that CPAP was largely performed by a small number of specialists, five key service requirement procedures remain. This is reflected in the number of procedures that SHOs and SpRs are "confident" or "very confident" to perform, and in the procedures which = 50% of SpRs and consultants identify as key generic skills for all. The limited exposure available to enable training, and the relatively small service requirement support a view that the PMETB requirement for all trainees to demonstrate competency in the remaining procedures (CPAP/BiPAP, tracheostomy management, knee joint aspiration, DC cardioversion and temporary pacing) is unachievable and unnecessary.

In keeping with previous studies, logistic regression identified the number of procedures performed as a key determinant of procedural confidence. Procedures performed within the past 12 months exhibited a greater effect on confidence than those previous. Male gender was also associated with increased confidence. There was no independent effect observed from skills lab based training methods, however the number of responders trained in this way was low resulting in a high possibility of type II error. No independent effect was observed for grade, years of clinical practice, or specialty.

In summary, doctors in hospital practice have a service requirement to provide five common practical procedures (central line insertion, lumbar puncture, pleural aspiration, ascitic aspiration, and intercostal drain insertion). This workload is currently being split between consultants and trainees, with trainees able to achieve confidence in these procedures before completion of training. Requirements for all trainees to achieve confidence in CPAP/BiPAP, tracheostomy management, knee joint aspiration, DC cardioversion, and temporary pacing are unachievable and unnecessary. Consultant practice is characterised by very high levels of confidence in a small number of procedures, with other procedures rarely if ever performed. Regaining and maintaining consultant confidence in other practical procedures would require ongoing performance of those procedures. This is unlikely to be achievable due to existing service commitments. The consequence of a shorter (run-through) training scheme is therefore predicted to be a decrease in the confidence of the practitioners who perform necessary practical procedures.

## Authors' contributions

RMC participated in the study coordination, statistical analysis and manuscript preparation. PC participated in the statistical analysis and manuscript preparation. PT participated in study coordination. AEK participated in the design of the study, obtained audit approval and participated in study coordination. EGK conceived the study, obtained audit approval and participated in study coordination.

## Pre-publication history

The pre-publication history for this paper can be accessed here:



## Supplementary Material

Additional file 1 **Current and previous UK system of medical training.** This figure shows the current and previous systems of medical training in the United Kingdom. Click here for file

Additional file 2 **Overview of procedural confidence responses.** This shows all responses for confidence in performing any procedure. Click here for file

Additional file 3 **Perception of further training requirement by grade.** This shows the percentage of all responses at each grade indicating a perceived need for further procedural training Click here for file

Additional file 4 **Perception that procedural competency for all doctors is important by grade.** This data combines responses for all procedures. It shows the percent of doctors at each grade rating specific procedures as important for all doctors to be competent in performing. Click here for file
