# Supplementary figures and images for: Procedural confidence in hospital based practitioners: implications for the training and practice of doctors at all grades
Source: BMC Med Educ. 2009 Jan 12;9:2. doi: 10.1186/1472-6920-9-2 (PMC2651872; doi:10.1186/1472-6920-9-2)

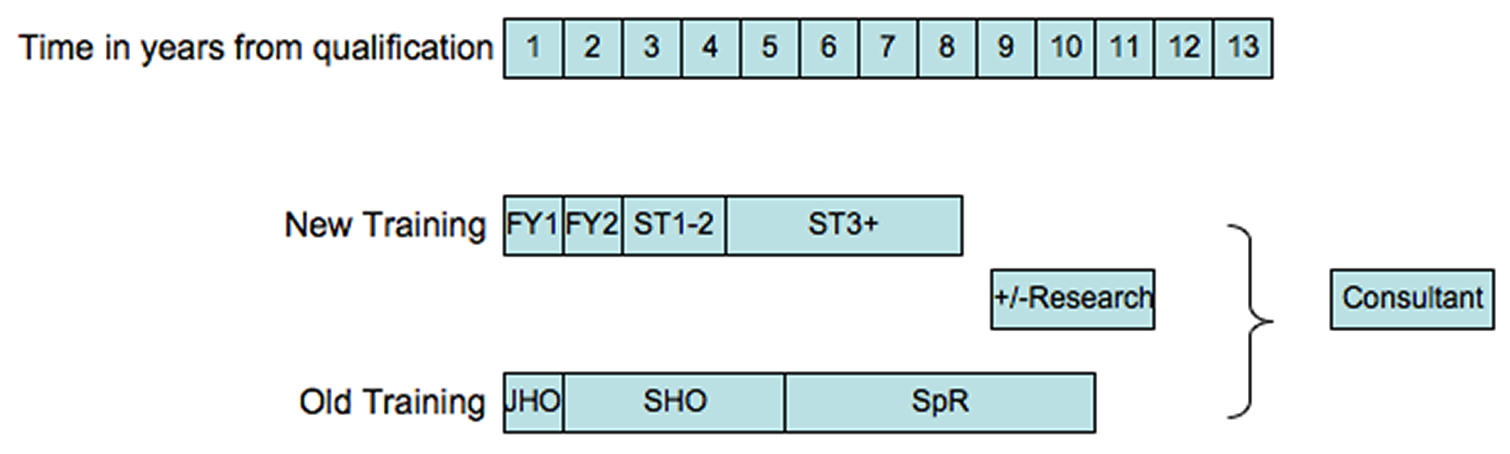

Supplement: Additional file 1 — Current and previous UK system of medical training. This figure shows the current and previous systems of medical training in the United Kingdom. [file 1472-6920-9-2-S1.tiff]

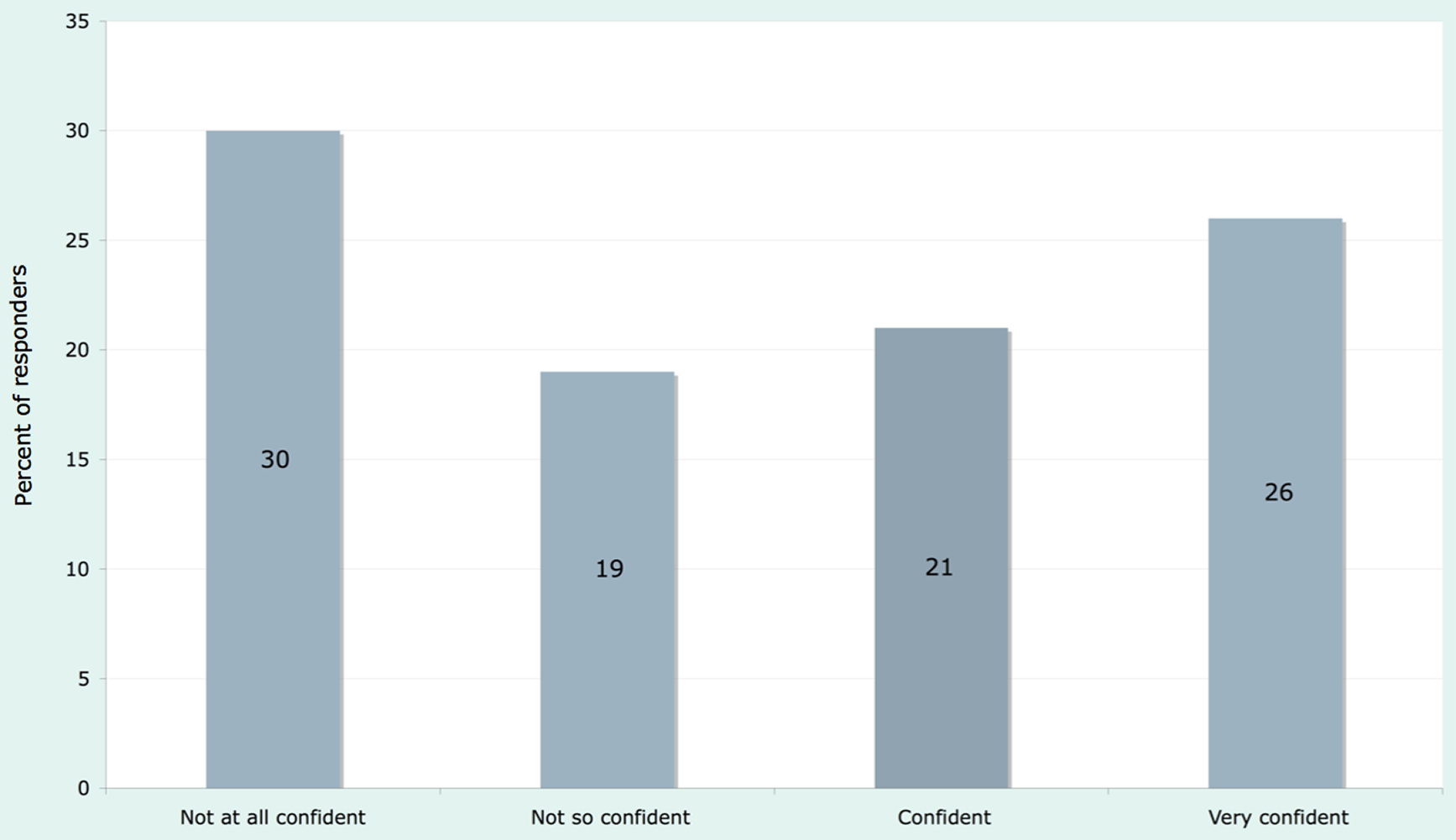

Supplement: Additional file 2 — Overview of procedural confidence responses. This shows all responses for confidence in performing any procedure. [file 1472-6920-9-2-S2.tiff]

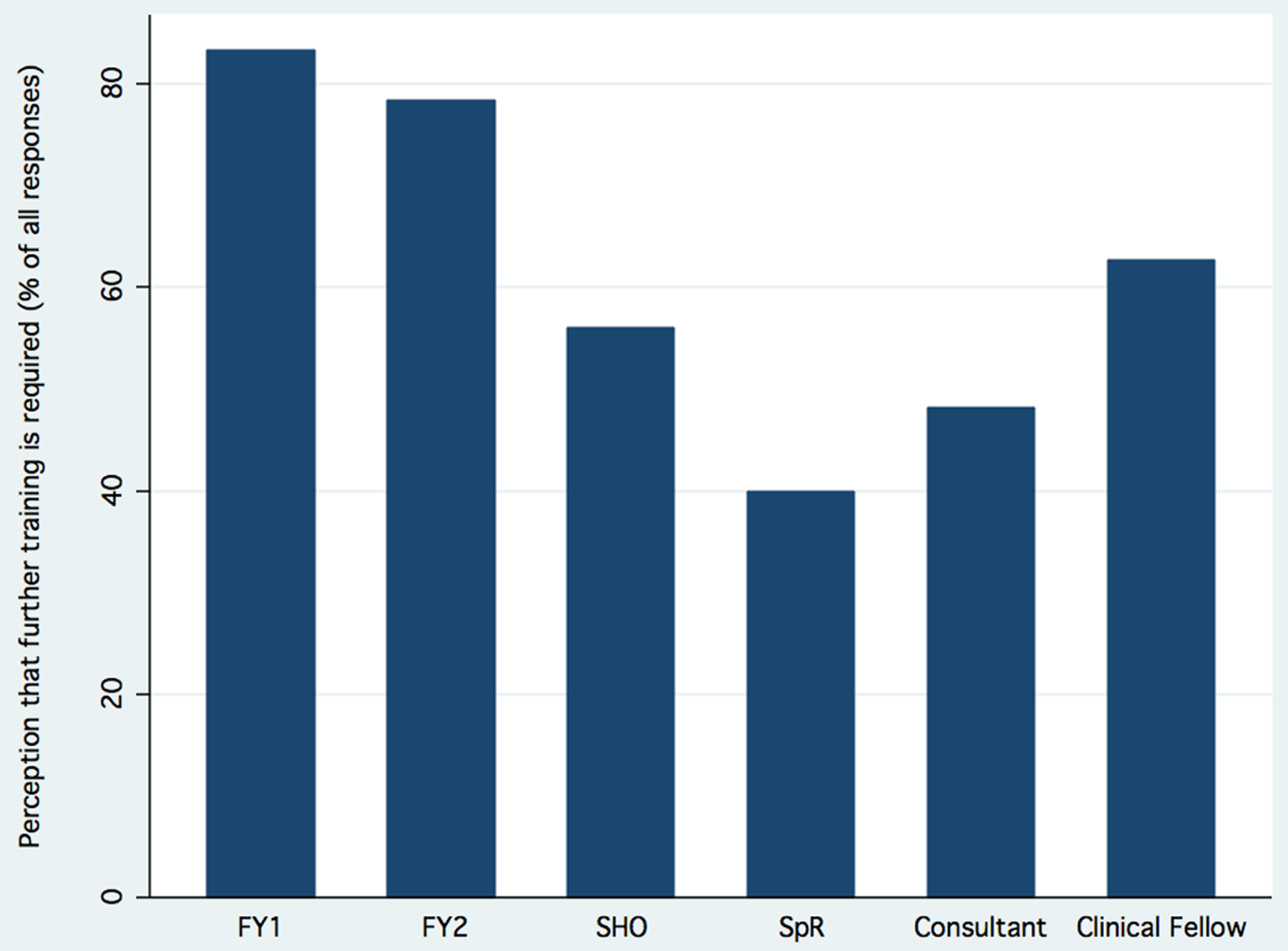

Supplement: Additional file 3 — Perception of further training requirement by grade. This shows the percentage of all responses at each grade indicating a perceived need for further procedural training [file 1472-6920-9-2-S3.tiff]

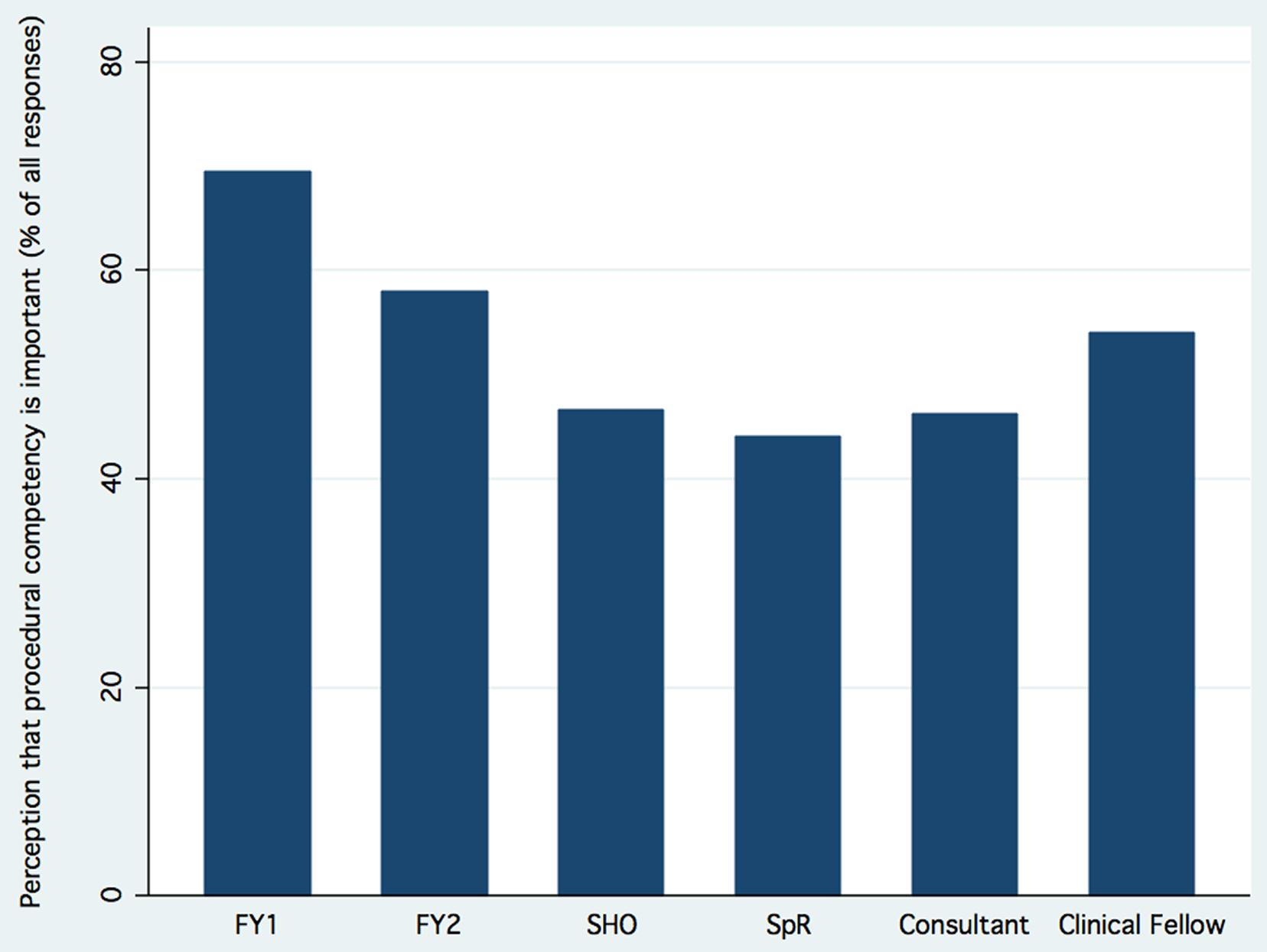

Supplement: Additional file 4 — Perception that procedural competency for all doctors is important by grade. This data combines responses for all procedures. It shows the percent of doctors at each grade rating specific procedures as important for all doctors to be competent in performing. [file 1472-6920-9-2-S4.tiff]
